# Pedunculated focal nodular hyperplasia of the liver in a healthy child born following in vitro fertilization: a case report and review of the literature

**DOI:** 10.1186/s13256-024-04512-4

**Published:** 2024-04-15

**Authors:** Mani Moayerifar, Pirouz Samidoust, Mahboobeh Gholipour, Maziar Moayerifar, Athar Zamani, Niloofar Poorheravi, Selvana Poursadrolah

**Affiliations:** 1 Razi Clinical Research Development Unit, Razi Hospital, Guilan University of Medical Sciences, Rasht, Iran; 2 Department of Cardiology, Healthy Heart Research Center, Heshmat Hospital, School of Medicine, Guilan University Of Medical Sciences, Rasht, Iran; 3 Department of Vascular Surgery, Razi Hospital, Guilan University of Medical Sciences, Rasht, Iran; 4 Department of Pathology and Laboratory Medicine, Guilan University of Medical Sciences, Rasht, Iran; 5 Student Research Committee, School of Medicine, Guilan University of Medical Sciences, Rasht, Iran

**Keywords:** Focal nodular hyperplasia, Pedunculated, Exophytic, Projected, In vitro fertilization, Child

## Abstract

**Background:**

Focal nodular hyperplasia is a common nonmalignant liver mass. This nonvascular lesion is an uncommon mass in children, especially those with no predisposing factors, namely radiation, chemotherapy, and hematopoietic stem cell therapy. Exophytic growth of the lesion further than the liver margins is not common and can complicate the diagnosis of the lesion. This report observes a focal nodular hyperplasia as a pedunculated lesion in a healthy child.

**Case presentation:**

We describe a 9-year-old healthy Persian child who was born following in vitro fertilization complaining of abdominal pain lasting for months and palpitation. Employing ultrasound and computed tomography, a mass was detected in the right upper quadrant compatible with focal nodular hyperplasia imaging features. The child underwent surgery and the mass was resected.

**Conclusion:**

Diagnosing focal nodular hyperplasia, especially pedunculated form can be challenging, although magnetic resonance imaging with scintigraphy is nearly 100% sensitive and specific. Thus, a biopsy may be needed to rule out malignancies in some cases. Deterministic treatment in patients with suspicious mass, remarkable growth of lesion in serial examination, and persistent symptoms, such as pain, is resection, which can be done open or laparoscopic.

## Background

Focal nodular hyperplasia (FNH) appears in 3% of the population and is usually observed in women aged 20–50 years. However, it can also affect males and individuals of other ages. The benign mass regularly appears solitary in the right lobe of the liver. Changes in sinusoidal pressure and perfusion can induce reactive hyperplasia in the liver parenchyma, leading to FNH formation. Therefore, studies have shown a greater incidence of FNH in patients with vascular malformations; however, it is also seen in healthy patients without any predisposing factors [1, 2, 4]. Among previously reported cases, some mentioned a chronic usage of oral contraceptives in patients.

These patients are mostly asymptomatic, and the lesions are detected incidentally. Occurrence of FNH in children is rare; however, case reports show possible connections between the lesion and celiac disease, anticardiolipin or antiphospholipid antibodies, and hematologic malignancies [3]. Pedunculated FNH, accounting for 9% of all FNH cases, is a rare manifestation that presents with an exophytic growth attached to the liver by a pedicle and makes it challenging to diagnose the lesion. While FNHs constitute only 2–7% of all hepatic tumors in children, we experienced an extensive pedunculated focal nodular hyperplasia in a healthy 9-year-old girl [4]. This article intends to emphasize the occurrence of FNH in healthy children and discuss the possible role of in vitro fertilization (IVF).

## Case presentation

**Fig. 1:**
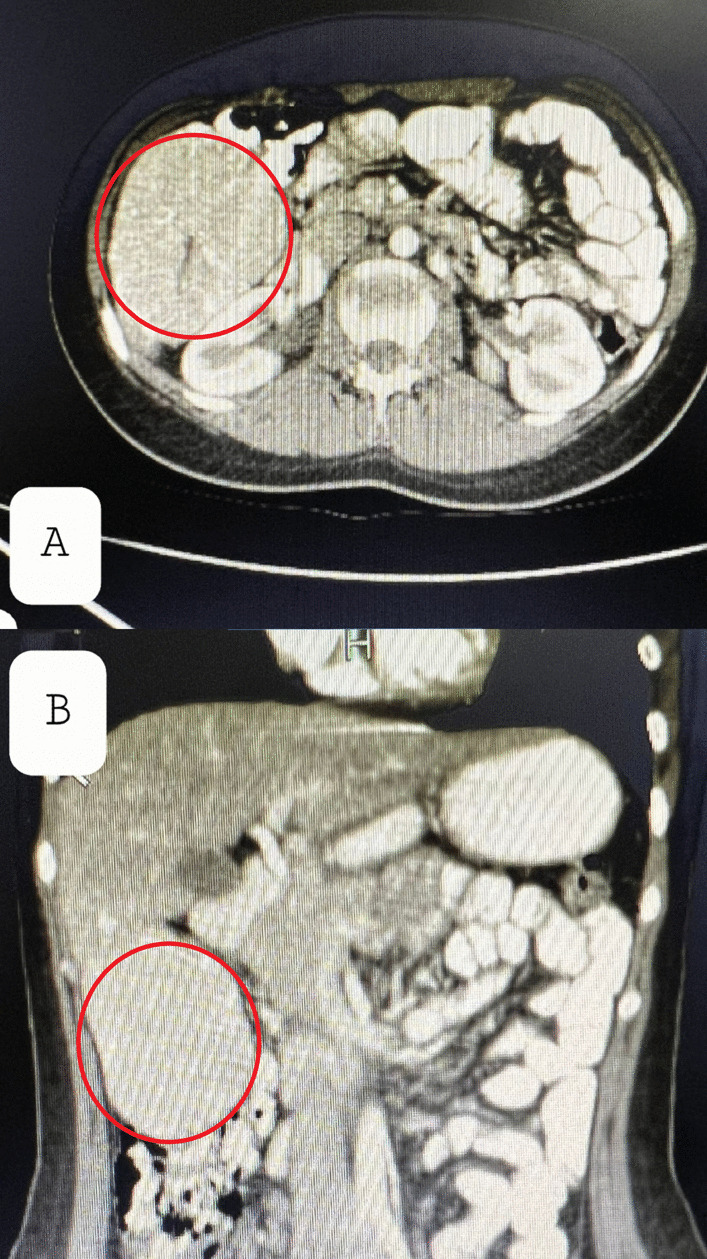
Computed tomography images. The red circles represent the FNH, which is demonstrated as a well-circumscribed mass emanating from the right lobe of the liver on axial (**A**) and coronal (**B**) planes

**Fig. 2:**
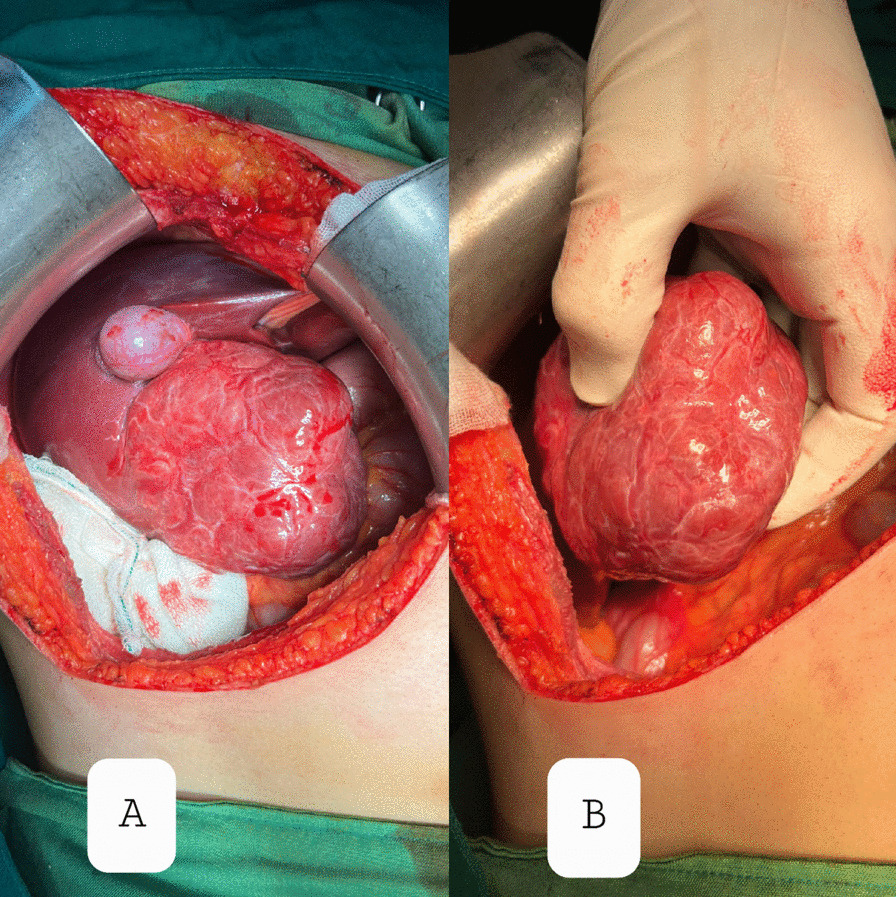
Intraoperative images. **A** Intraoperative picture of a large pedunculated mass originating from the right lobe of the liver. **B** Mass resection

**Fig. 3:**
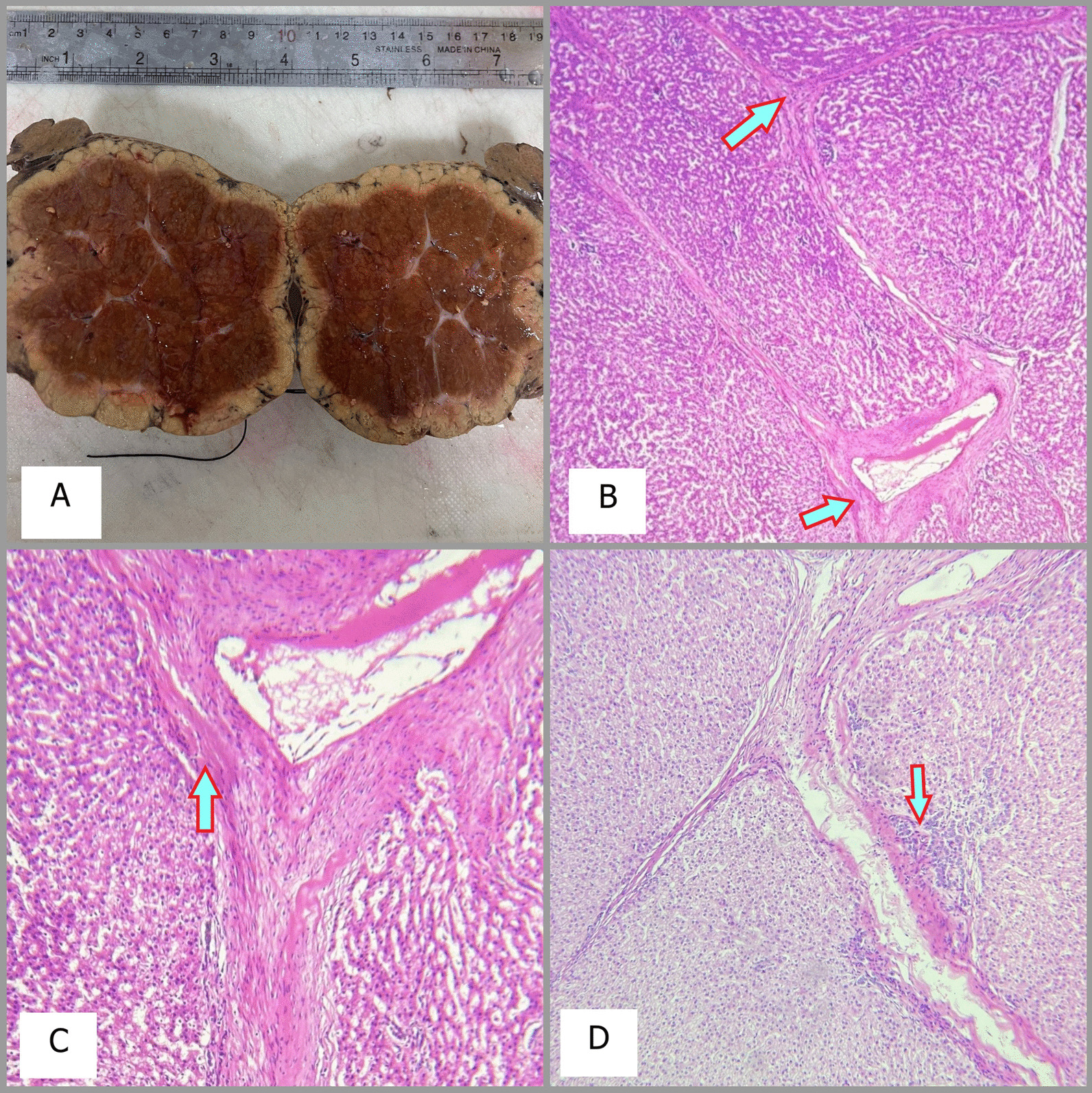
Macroscopic and microscopic view of the mass. Macroscopic and microscopic view of the mass. **A** A well-demarcated, solitary brown–orange lesion with a central scar. **B** Focal nodular hyperplasia is composed of bland hepatocytes surrounded by fibrous septa, pointed by arrows, containing artery branches. **C** A med large-sized, thick walled muscular vessel with hyperplastic changes is demonstrated by the arrow. **D** The arrow points to ductular reaction that is a characteristic feature of focal nodular hyperplasia

A 9-year-old Persian girl complaining of burning pain in the epigastrium, palpitations, and no other symptoms presented to our department. The nonpositional pain started 6 months before our visit and was not related to feeding or transit. She was referred to us by a cardiologist after a palpable mass in the right upper quadrant (RUQ) was detected. She did not mention any drug usage, allergies, or past medical or family history except the fact that she was born as a result of in vitro fertilization (IVF). On the physical examination and routine laboratory tests, which are reported below in the box, the only noticeable finding was a mild bulging in the RUQ. We performed an ultrasonography (US), demonstrating a large echogenic mass with moderate vascularity adjacent to the segment VI of the liver. A few days later, on the computed tomography (CT), a large, well-circumscribed mass (92 mm × 70 mm) without hemorrhage, hypervascular in portal phase with a central nonenhanced scar was present and seemed to emanate from the right liver lobe (Fig. 1). The lesion was hypodense in the precontrast phase and isoattenuated to the liver parenchyma in the equilibrium phase. The fibrotic scar demonstrated enhancement on the delayed scan. Based on the CT findings, pedunculated liver focal nodular hyperplasia and hepatocellular adenoma were considered. We checked for alpha fetoprotein and antihydatid antibody levels, which were both normal. For a decisive diagnosis and histopathological examination, the patient underwent surgery. Opening the abdomen, a large lesion was observed below the right lobe of the liver (Fig. 2). The lesion was resected, and macroscopic evaluation of the liver mass revealed a well-demarcated, solitary brown‒orange lesion with a central scar (Fig. 3A). No evidence of recurrence was detected on examination and US at the 1 month follow-up. Microscopic sections revealed a well-differentiated hepatocellular lesion with fibrous septa containing artery branches, a ductular reaction, and mixed inflammation. Nuclear pleomorphism, prominent nucleoli, and mitotic figures were absent (Fig. 3B–D). The above-mentioned morphologic features suggested FNH.

## Discussion

FNH is a benign lesion accounting for 8% of all nonhemangiomatous liver masses. The exact etiology of the lesion is still under debate, but it seems that hypoperfusion or hyperperfusion in the liver due to arterial malformation and changes in perfusion can cause a regenerative hyperplastic response in normal hepatocytes, leading to FNH [1]. FNH is often asymptomatic but can present as abdominal pain or a tender abdominal mass, especially when the lesion is larger than 10 cm [1, 5]. Same as our study, a few cases reported an uncommon pedunculated lesion of the liver, which is susceptible to torsion or becoming symptomatic and causing intraperitoneal hemorrhage. Navarini *et al*. reported a 26-year-old female presenting with an acute abdomen caused by pedicle torsion of a pedunculated FNH [6]. FNH is mainly reported in children with certain risk factors, such as chemotherapy, hematologic cancers, underlying liver disease, and history of radiotherapy; however, a previous case reported a healthy child with no medical history but who was born prematurely [7]. We experienced FNH in a child who was born following IVF, and we assume that there might be a correlation between IVF and FNH in a healthy child. Identical to the controversial effect of oral contraceptives on FNH, which is still not definite, more investigation is needed to find the possible connection between IVF and FNH [8–10].

Most often, laboratory data do not provide a conclusive diagnosis of FNH. However, there might be a slight rise in gamma-glutamyl transferase levels in some cases [4]. US is usually the initial imaging method with low sensitivity, performed when doubting FNH. However, a previous study showed a 96% success rate for contrast-enhancing US in ruling out hepatic adenoma. The US demonstrates an isoechoic or hypoechoic mass with a hyperechoic central scar relative to the liver parenchyma. Another widely used modality is triphasic helical CT with and without contrast agent, in which lesions appear hypodense or isodense before contrast agent administration, hyperdense during the arterial phase, and hypodense during the venous phase. The magnetic resonance imaging with hepatocyte scintigraphy with a sensitivity and specificity of 99% and 100% is the most accurate imaging modality for diagnosing the FNH. Using hepatocyte specific contrast agents, such as gadobenate dimeglumine, makes the lesion hyperintense relative to the normal liver parenchyma, which is particularly common in FNH [1, 7, 11]. Even using the imaging modalities, due to challenges in diagnosing FNH, especially the pedunculated form, sometimes biopsy may be needed. For instance, in a 35-year-old female, fine needle aspiration ruled out a gastrointestinal stromal tumor for a suspicious perigastric mass detected by CT [12–14].

At the time of presentation, our patient had an US revealing a hepatic mass. Due to the normal laboratory tests, tumor markers, the patient’s age, and imaging features of the lesion described earlier, malignant lesions were ruled out, and the mass was highly suspected to be FNH or hepatocellular adenoma, which the biopsy defined the mass to be FNH.

In asymptomatic, incidentally detected patients with FNH, only serial imaging is enough. In contrast, in pedunculated lesions at risk of rupture, torsion, intraperitoneal hemorrhage, continuous growth of the lesion, and becoming symptomatic, the patient should undergo surgery or biopsy [1, 3, 5]. Among the cases, one suggested a conservative management of an exophytic FNH [15], while another one described a rupture of projected FNH 3 years after the initial diagnosis, leading to resection of segment IV/V of the liver [16]. Before surgery, hepatic angiography may be conducted to reduce the vascularity of the tumor, as was done in the case reported by Khan *et al*. [17].

In a macroscopic view, the central scar is remarkable in FNHs and consists of mature collagen surrounded by vessels and fibrous septa; however, it may be absent in some cases. In microscopic view, multiple nodules of hepatocytes with granular cytoplasm between the fibrous septa can be seen. Bile ducts and Kupffer cells may also be present. Both hematoxylin and eosineosin (H&E) and reticulin stains can be helpful in diagnosing the tissue accurately [1–3, 7, 12]. A summary of the previously reported cases of the pedunculated FNH is presented in Table 1.

**Table 1:** Summary of the previous literature in pedunculated focal nodular hyperplasia

Author/year of publication	Sex/age (years)	Symptoms	Size	Location	Laboratory	Imaging	Treatment	Follow-up	Chronic OCP usage
Sawhney *et al*. (1991) [18]	Female/12	Asymptomatic	N/A	Left lobe of the liver	N/A	US and CT	Surgery	N/A	No
Byrnes *et al*. (2004) [9]	Female/30	RUQ pain	Multiple lesions (the largest one was 16 cm × 18 cm)	Seg 3, 4B	Normal	Noncontrast MRI and CT angiogram	Surgery	No complications	No But she was pregnant
Wasif *et al*. (2008) [2]	Female/48	RUQ pain	3.2 cm × 2.4 cm × 2.4 cm	Seg 4B	Normal	US and CT	Surgery	No recurrence	No
Khan *et al*. (2011) [17]	Female/29	Abdominal pain and emesis	19.5 cm × 15.8 cm × 9.5 cm	Seg 3, 4B	Anemia and elevated liver enzymes	CT	Surgery	No recurrence	No
Badea *et al*. (2014) [14]	Female/29	Abdominal pain	8 cm × 5 cm	Seg 5	Normal	US, CEUS, and CT	Laparoscopic resection of the mass and gall bladder	No complications	Yes
Reddy *et al*. (2015) [12]	Female/35	Chronic abdominal pain	2.89 cm × 2.7 cm	Seg 3	N/A	CT and EUS	Laparoscopic resection	N/A	N/A
Zeina *et al*. (2016) [15]	Female/25	Epigastric pain and nausea	4.8 cm	Seg 2	Normal	US, CT, and MRI with scintigraphy using gadobenate dimeglumine	N/A	N/A	No
Zhuang *et al*. (2016) [19]	Male/6	Asymptomatic	10.5 cm × 9.9 cm	Right hepatic lobe	Normal	US and CT	Surgery	No complications	No
Kinoshita *et al*. (2017) [16]	Male/32	Asymptomatic	8 cm	Seg 4, 5	Elevated CRP, WBC, AST, and ALT	CT, MRI, and in the readmission due to rupture of the lesion a hepatic angiography was also done	Conservative management at first but due to rupture of the lesion patient underwent surgery	No complication after surgery	No
Koolwal *et al*. (2018) [7]	Female/3	Abdominal distension	5.9 cm	Right hepatic lobe	Normal	US, CT, and MRI	Surgery	N/A	No (history of preterm birth)
Martiniuc *et al*. (2018) [13]	Female/40	Asymptomatic	4.2 cm × 5 cm	Seg 3	Normal	US and contrast enhanced CT	Surgery	No complications	No
Navarini *et al*. (2020) [6]	Female/26	Acute abdomen and a palpable mass in the epigastrium	13 cm × 7 cm	Left hepatic lobe	N/A	MRI	Surgery	N/A	N/A
Alfayez *et al*. (2021) [5]	Male/5	RUQ pain	9 cm × 10 cm × 12 cm	Seg 5, 8	Normal	US, CT, and MRI	Surgery	No complications	No
Ismail *et al*. 2021 [10]	Female/38	Asymptomatic	4.5 cm × 3.5 cm × 3 cm	Seg 3	Normal	N/A (detected intraoperatively during surgical repair of hernia)	Surgery	No recurrence	Yes
Akaguma *et al*. (2023) [20]	Female/35	RUQ abdominal pain	7 cm × 7 cm × 5 cm	Seg 6	N/A	US and CT	Surgery	No complication	Yes

*OCP oral contraceptives, N/A* not answered, *Seg* segment, *US* ultrasonography, *CT* computed tomography, *RUQ* right upper quadrant, *MRI* magnetic resonance imaging, *CEUS* contrast enhanced ultrasound, *EUS* endoscopic ultrasound, *CRP* c-reactive protein, *WBC* white blood cell, *AST* aspartate aminotransferase, *ALT* alanine aminotransferase

## Conclusion

FNH, a common mass that appears in the liver in adulthood, can present as an exophytic pedunculated mass that increases the risk of intraperitoneal hemorrhage and rupture and can lead to misdiagnosis of the lesion as an extrahepatic mass, such as a gastrointestinal stromal tumor. Thus, surgical resection of the tumor is the treatment of the choice. Moreover, although rare, pedunculated FNH is a considerable differential diagnosis even in healthy children without any past medical history, presenting with epigastric or RUQ pain or tenderness.

## Data Availability

Data supporting this article are available in Razi Hospital Data center, Rasht, Iran.

## Abbreviations

FNH: Focal nodular hyperplasia
IVF: In vitro fertilization
RUQ: Right upper quadrant
US: Ultrasonography
CT: Computed tomography
